# Utility of Huntington's Disease Assessments by Disease Stage: Floor/Ceiling Effects

**DOI:** 10.3389/fneur.2021.595679

**Published:** 2021-07-15

**Authors:** Daisy Abreu, Jennifer Ware, Nellie Georgiou-Karistianis, Blair R. Leavitt, Cheryl J. Fitzer-Attas, Raquel Lobo, Ana Raquel Fernandes, Olivia Handley, Karen E. Anderson, Julie C. Stout, Cristina Sampaio

**Affiliations:** ^1^Associação para Investigação e Desenvolvimento da Faculdade de Medicina, Universidade de Lisboa, Lisbon, Portugal; ^2^CHDI Management/CHDI Foundation, Princeton, NJ, United States; ^3^Turner Institute of Brain and Mental Health, School of Psychological Sciences, Monash University, Melbourne, VIC, Australia; ^4^Centre for Molecular Medicine and Therapeutics, Department of Medical Genetics, University of British Columbia, Vancouver, BC, Canada; ^5^ClinMed LLC, Dayton, NJ, United States; ^6^School of Biosciences, Cardiff University, Cardiff, United Kingdom; ^7^Department of Psychiatry and Department of Neurology, Georgetown University, Washington, DC, United States

**Keywords:** Huntington's disease, clinimetrics properties, utility of measurements, clinical assessments, Enroll-HD

## Abstract

**Introduction:** An understanding of the clinimetric properties of clinical assessments, including their constraints, is critical to sound clinical study and trial design. Utilizing data from Enroll-HD—a global, prospective HD observational study and clinical research platform—we examined several well-established HD clinical assessments across all stages of disease for evidence of instrument constraints, specifically floor/ceiling effects, to inform selection of appropriate instruments for use in future studies/trials and identify gaps in instrument utility over the life-course of the disease.

**Material and Methods:** Analyzing publicly available data from 6,614 HD gene-expansion carriers (HDGECs), we grouped participants into deciles based on baseline CAP score, which ranged from 26 to 229. We used descriptive statistics to characterize data distribution for 25 outcome measures (encompassing motor, function, cognition, and psychiatric/behavioral domains) in each CAP decile. A skewness statistic threshold of ±2 was defined *a priori* to indicate floor/ceiling effects.

**Results:** We found evidence of floor/ceiling effects in the early premanifest stages of disease for most motor and function assessments (e.g., TMS, TFC) and select cognitive tasks (MMSE, Trail Making tests). Other cognitive assessments, and the HADS-SIS scales, performed well ubiquitously, with no evidence of floor/ceiling effects at any disease stage. Floor/ceiling effects were evident at every disease stage for certain assessments, including PBA-s measures. Ceiling effects were apparent for DCL from onset stages onwards, as expected.

**Discussion:** Developing instruments sensitive to subtle differences in performance at the earlier stages of the disease spectrum, particularly in motor and function domains, is warranted.

## Introduction

Huntington's disease (HD) is an autosomal dominant, progressive, neurodegenerative disease characterized by debilitating movement, cognitive and psychiatric disturbances (1). It is caused by a mutation in the CAG repeat region of the *HTT* gene, defined by the presence of ≥36 CAG repeats. Clinical diagnosis of HD is typically based on the unequivocal presence of extrapyramidal motor signs. Onset most commonly occurs in mid-adulthood, although subtle cognitive, motor and psychiatric symptoms may be detected many years prior to formal clinical diagnosis (2). Symptoms progressively worsen post-onset, leading to death, typically within 10–30 years of diagnosis (3).

Several now well-established clinical assessments have been developed to assess and track the evolution of HD symptomology over time. Critically, these assessments measure impairments in one or more critical disease domains: motor, function, cognition and psychiatric/behavioral. Given the progressive nature of HD, it follows that the utility of these assessments may vary by disease stage/severity, dependent on instrument design, including range and sensitivity constraints.

Here we focus on ceiling and floor effects, which are commonly observed phenomena in data, particularly in clinimetric contexts. “Ceiling effect” describes a situation in which many values for a given variable are at or near the upper limit (ceiling) of the scale used to measure said variable (4). Distributions of values in such situations will typically be very heavily skewed and variance limited, which can prove problematic for many types of analyses and give rise to spurious conclusions. Conversely, “floor effect” causes similar problems.

Utilizing publicly-available data from Enroll-HD—a worldwide prospective observational study and clinical research platform—we sought to identify floor/ceiling effects for the most common clinical measures used in HD research at different stages of disease across the full life course.

## Materials and Methods

### Study Population

#### Enroll-HD

Enroll-HD (https://www.enroll-hd.org/) is a prospective cohort study and global clinical research platform designed to facilitate clinical research in HD (5, 6). Enroll-HD encompasses over 150 sites, from 19 countries located in North America, Latin America, Europe, Australia and New Zealand. Data are collected from participants annually and are monitored for quality and accuracy using a risk-based monitoring approach. Sites are required to obtain and maintain local Ethics Committee approvals.

#### Analysis Dataset

The third Enroll-HD periodic dataset, released December 15, 2016, was used for analysis. Analyses were limited to cross-sectional Enroll-HD baseline visit data from HD gene expanded carriers (HDGECs) only.

The Enroll-HD periodic dataset is available to any interested researcher for download through the Enroll-HD website (https://www.enroll-hd.org/for-researchers/access-data/).

#### Participants and Visits

A maximum of 6,614 participants were available for analysis. Table 1 provides an overview of participant demographic and disease stage characteristics.

**Table 1:** Participant characteristics at baseline visit (global and stratified by disease stage).

	***N***	**Male**	**Age (years)**	**Post-high school education**	**CAG length**
		***N* (%)**	**Mean (SD)**	***N* (%)**	**Mean (SD)**
Total participants	6,614	3,071 (46.0%)	49.4 (13.7)	3,112 (47.3%)	43.6 (3.8)
TFC stage^*^					
Pre-manifest	1,862	729 (39.2%)	40.4 (12.0)	1,138 (61.1%)	42.4 (2.8)
Manifest I (TFC 11–13)	1,365	730 (53.5%)	50.0 (12.0)	681 (50.1%)	43.6 (3.5)
Manifest II (TFC 7–10)	1,717	851 (49.6%)	52.8 (12.5)	726 (42.3%)	43.9 (3.8)
Manifest III (TFC 3–6)	1,123	527 (46.9%)	54.9 (12.7)	414 (36.9%)	44.4 (4.2)
Manifest IV (TFC 1–2)	390	168 (43.1%)	56.2 (13.1)	112 (29.0%)	45.0 (5.1)
Manifest V (TFC 0)	151	64 (42.4%)	55.7 (12.4)	38 (27.3%)	45.7 (4.2)
**CAP score decile**					
CAP 1 (25.89–66.26)	682	420 (61.6%)	31.4 (8.4)	406 (59.5%)	41.9 (2.8)
CAP 2 (66.26–79.50)	650	390 (60.0%)	40.3 (10.0)	374 (57.6%)	42.5 (3.1)
CAP 3 (79.51–88.75)	656	373 (56.9%)	44.1 (9.6)	330 (50.3%)	43.1 (3.3)
CAP 4 (89.06–97.07)	723	393 (54.4%)	47.2 (10.3)	381 (52.8%)	43.6 (3.4)
CAP 5 (98.00–101.69)	526	265 (50.4%)	50.3 (10.0)	246 (46.9%)	43.5 (3.3)
CAP 6 (102.16–107.40)	701	348 (49.7%)	51.7 (10.5)	290 (41.6%)	43.9 (4.1)
CAP 7 (107.86–111.86)	586	277 (47.3%)	54.1 (11.2)	267 (45.6%)	43.8 (3.4)
CAP 8 (112.17–117.1)	702	363 (51.7%)	56.7 (10.8)	282 (40.5%)	43.7 (3.3)
CAP 9 (117.87–125.42)	688	344 (50.0%)	58.1 (11.6)	302 (44.2%)	44.2 (3.6)
CAP 10 (125.73–228.66)	700	370 (52.9%)	59.5 (13.8)	234 (33.9%)	45.8 (5.3)

* *TFC score missing for 6 participants prohibiting determination of TFC stage*.

### Variables

#### Clinical Assessments and Outcomes

The Enroll-HD clinical assessment battery includes assessments for motor, function, cognition, and psychiatric/behavioral domains. These assessments are administered by a trained rater in a clinic setting. Certain Enroll-HD assessments are administered as standard at each study visit (“core” assessments), while others are completed at the discretion of the site investigator (“extended” assessments; denoted in Table 2).

**Table 2:** Clinical assessments and outcome measures.

**Assessment**	**Outcome variable**	**Domain**	**Range**		**Direction of scoring(Higher scores)**	**References**
			**Min**	**Max**		
UHDRS Motor (TMS)	Motor score	Motor	0	124	Worse	(7)
UHDRS Motor/ Diagnostic Confidence (DCL)	Diagnostic confidence level	Motor	0	4	Increased confidence in motoric onset	(8)
UHDRS Total Functional Capacity (TFC)	Functional score	Function	0	13	Better	(9)
UHDRS Function Assessment (FAS)	Functional assessment score	Function	0	25	Better	(10)
UHDRS Independence Scale (IS)	Subject's independence in %	Function	5	100	Better	(11)
Physiotherapy Outcome—Timed “Up and Go” Measures (TUG)^*^	Total time	Motor	>0	N/A^a^	Worse	(12)
Symbol Digit Modality Test (SDMT)	Total correct	Cognition	0	110	Better	(13)
Verbal Fluency Test (Category)	Total correct (1 min)	Cognition	0	N/A	Better	(14)
Stroop Color Naming Test (SCNT)	Total correct	Cognition	0	N/A^b^	Better	(15)
Stroop Word Reading Test (SWRT)	Total correct	Cognition	0	N/A	Better	(16)
Stroop Interference Test (SIT)^*^	Total correct	Cognition	0	N/A	Better	(17)
Trail Making Test (TRLMT)^*^	Part A: time to complete	Cognition	>0	240	Worse	(18)
Trail Making Test (TRLMT)^*^	Part B: time to complete	Cognition	>0	240	Worse	(18)
Verbal Fluency Test (Letters)^*^	Total correct (3 min)	Cognition	0	N/A	Better	(14)
Mini Mental State Examination (MMSE)^*^	MMSE score	Cognition	0	30	Better	(19)
Problem Behaviors Assessment—Short (PBA-s)	Depression	Behavioral	0	48	Worse	(20)
	Irritability aggression	Behavioral	0	32	Worse	(21)
	Psychosis	Behavioral	0	32	Worse	(22)
	Apathy	Behavioral	0	16	Worse	(23)
	Executive function	Behavioral	0	32	Worse	(24)
Hospital Anxiety and Depression Scale—Snaith Irritability Scale (HADS-SIS)^*^	Anxiety subscore	Behavioral	0	21	Worse	(25)
	Depression subscore	Behavioral	0	21	Worse	(25)
	Irritability subscore	Behavioral	0	24	Worse	(26)
**Assessment**	**Outcome Variable**	**Domain**	**Range**		**Direction of Scoring(Higher Scores)**	**References**
			**Level**	**t-scores**		
Short Form Health Survey−12v2 (SF-12)^*^	Physical Functioning (PF)	Motor	1 2 3 4 5 6 7	55+ 50–54.9 45–49.9 40–44.9 35–39.9 30–34.9 <30	Better	(27)
	Mental Health (MH)	Behavioral	1 2 3 4 5 6 7 8 9	60+ 55–59.9 50–54.9 45–49.9 40–44.9 35–39.9 30–34.9 25–29.9 <25	Better	

* *Optional (“extended”) assessment in the Enroll-HD assessment battery*.

a *Observations >120 excluded (n = 2) per Enroll Quality Control procedure*;

b *Observations of >150 excluded (n = 1)*.

We focused on 25 commonly used outcome measures drawn from these assessments, as listed in Table 2. Given the optional nature of the “extended” assessments, analysis of these outcomes was based on a more limited sample relative to those outcomes from “core” assessments.

#### Gene Carrier Status

Analyses were limited to HDGECs, defined as individuals with a CAG length ≥36 as determined at a central laboratory (Biorep Technologies, Inc.).

#### Disease Stage/Severity

CAP score, derived from CAG length and age, is indicative of cumulative exposure to mutant huntingtin (akin to “pack/years” for assessing tobacco exposure in smokers), and was used to approximate disease stage/severity. CAP score was calculated based on the Warner^1^ formula, which is standardized to ensure that CAP = 100 at the expected age of diagnosis:

$$\begin{array}{l} \text{CAP score }=\text{ Age }*\left(\text{CAG}-\text{L}\right)/\text{K},\text{ where L}=30\text{ and K}=6.49 \end{array}$$

Participants were subdivided into deciles based on CAP score at baseline for the purposes of HD staging using the quantile function in R (quantile ()). This enabled approximation of a disease stage/severity gradient (CAP score decile 1 = least severe; 10 = most severe). Note that a CAP score of 100 fell within CAP decile 5 (Table 1). Participants were also characterized according to the Shoulson-Fahn I-V staging system (28) using Total Functional Capacity (TFC) assessment score (Table 1).

### Statistical Methods

Clinical outcome measures were characterized by CAP score deciles, using descriptive statistics of central tendency and variability to characterize data distribution (mean, standard deviation, maximum, minimum, skewness). A skewness statistic threshold of ±2 was defined *a priori* to indicate substantial departure from normality/extreme positive or negative skew, indicative of floor/ceiling effects (29, 30). In addition, a complementary method to assess such effects was applied in which the percentage of participants scoring minimum and maximum scores was calculated within each CAP score decile for each assessment with defined upper and lower score bounds.

Data points outside of minimum or maximum scale thresholds (see Table 2) were excluded (Trail Making Test part *B* = 17 observations; Trail Making Test part *A* = 2 observations), as were extreme outliers from assessments with no maximum score [Stroop Color Naming Test = 1 observation (scores ≥400); Time up and go = 2 observations (scores ≥120 s)]. Additional sensitivity analyses were performed *post-hoc* for assessments with no maximum score to evaluate the impact of outliers on skewness statistics.

Statistical analyses were performed using R version 3.0.3.

## Results

### Participant Characterization

**Figure 1:**
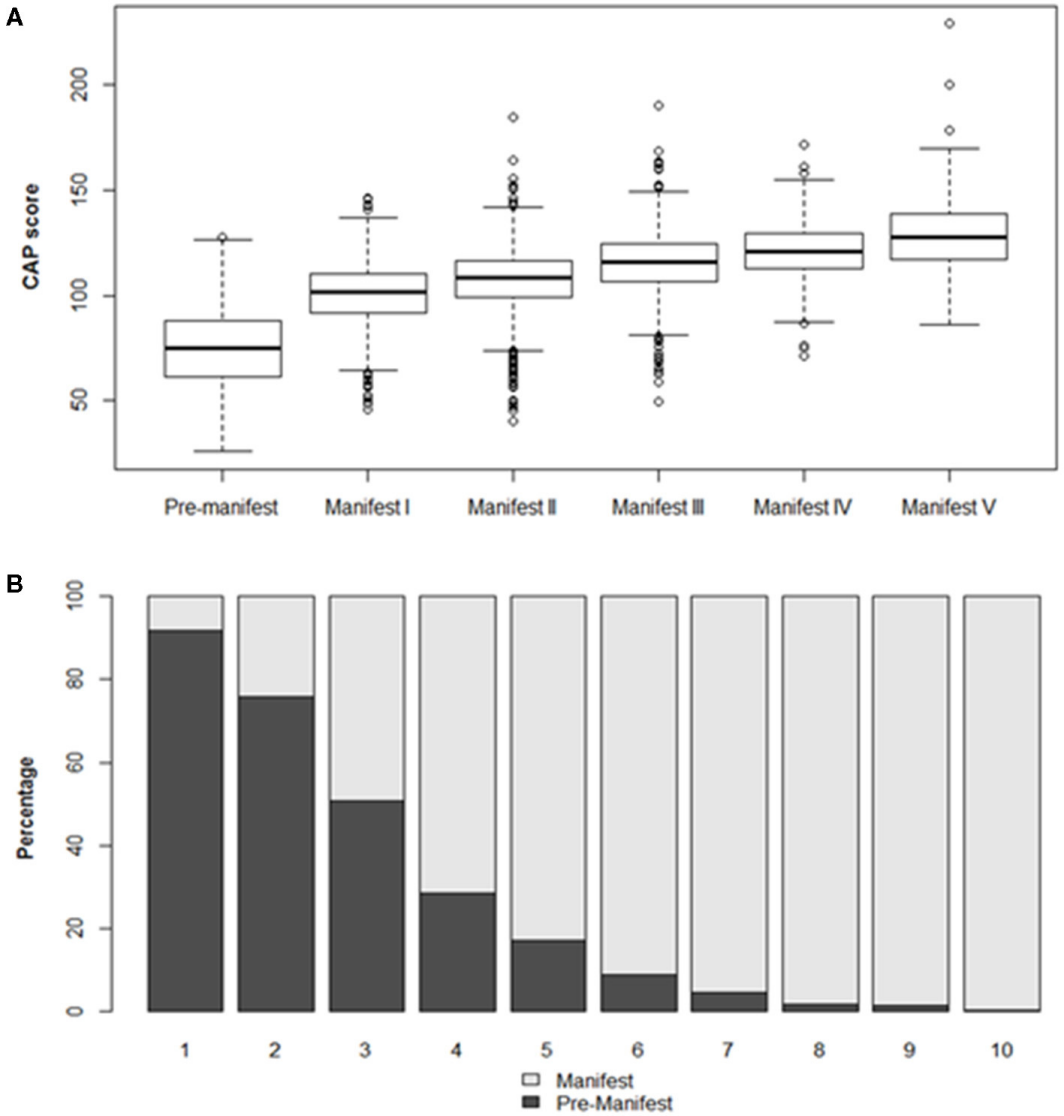
Participant characteristics by disease stage at baseline visit. **(A)** CAP score distribution by disease stage (TFC defined). Boxes indicate interquartile range (IQR), spanning the 25–75th percentile. Horizontal lines indicate median values. Whiskers indicate 25th percentile minus 1.5*IQR (lower) and 75th percentile plus 1.5*IQR (upper). Observations beyond these thresholds are indicated by open circles. **(B)** Manifest status by CAP score decile.

Participant demographic and disease stage characteristics, determined at Enroll-HD baseline visit, are presented in Table 1 and Figure 1.

### Clinical Outcome Characterization by Cap Score

**Figure 2:**
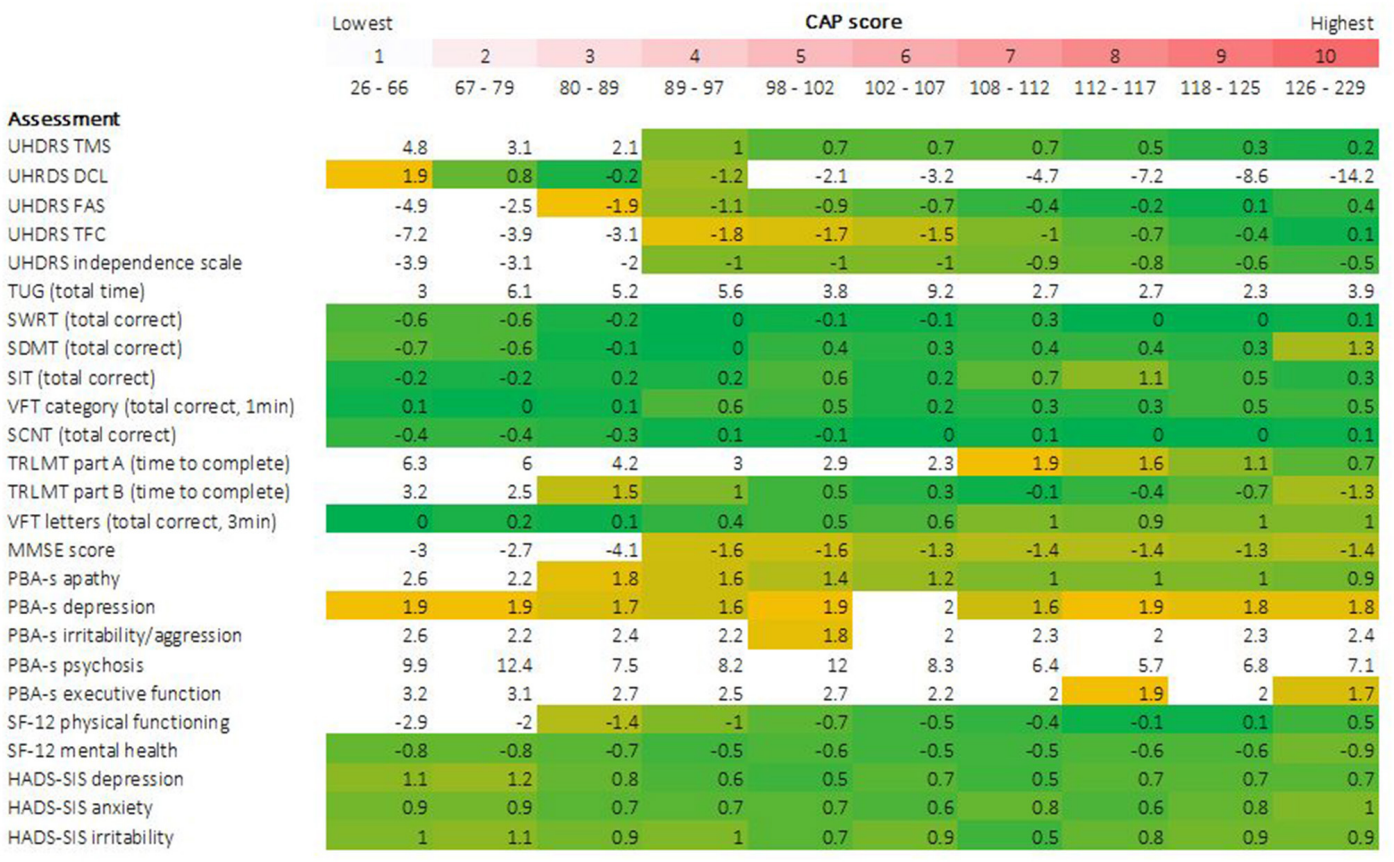
Skewness of clinical assessment data by CAP score. Degree of data skewness observed within each CAP score decile is illustrated for each clinical outcome. Cells are color coded conditional on observed skewness statistic. A threshold of ±2, indicating extreme positive or negative skew, was defined *a priori* to identify extreme skew, indicative of floor/ceiling effects. Cells with values more extreme than these thresholds are colored white. All remaining cells, with skewness statistics ranging from −2 to +2, are color coded on a yellow-green-yellow gradient, centered at 0 (green), indicative of a perfect normal distribution of data, graduating to yellow as values become more extreme.

**Figure 3:**
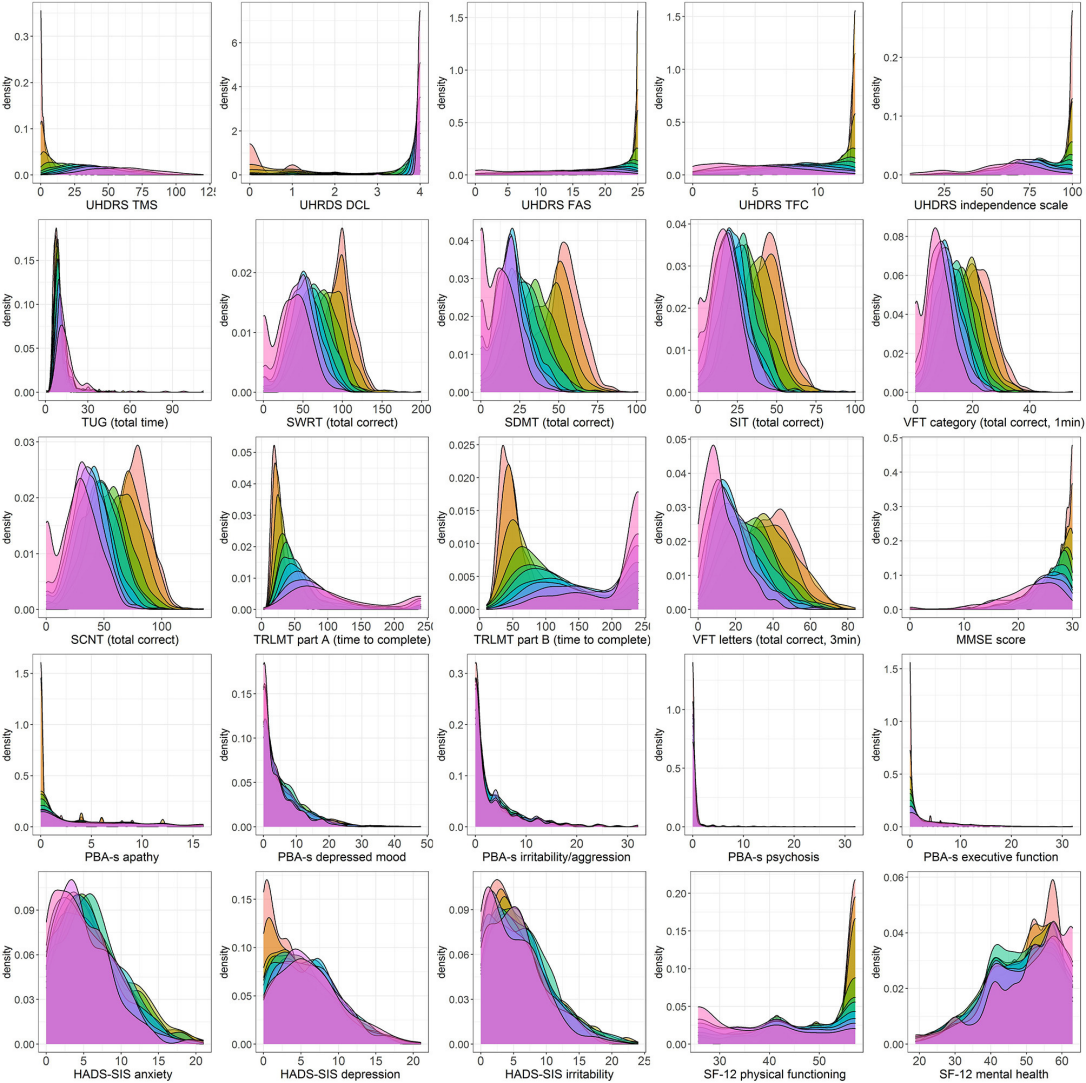
Density plots of clinical assessment data by CAP score. Color legend for each CAP decile: 
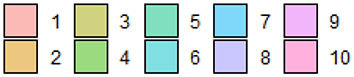
.

Descriptive statistics characterizing each outcome as a function of CAP score decile are presented in Table 3. Accompanying density plots, illustrating the observed distribution of data for each outcome as a function of CAP score decile, are presented in Figure 3. The degree of skewness observed for clinical outcome data in each CAP decile is illustrated in Figure 2. The percentage of participants scoring minimum and maximum scores within each CAP score decile for each assessment is presented in Supplementary Table 1.

**Table 3:** Clinical outcome measures by CAP score decile at baseline visit.

**Clinical scale**	**CAP 1**		**CAP 2**		**CAP 3**		**CAP 4**		**CAP 5**		**CAP 6**		**CAP 7**		**CAP 8**		**CAP 9**		**CAP 10**	
	**(25.89–66.26)**		**(66.56–79.04)**		**(79.51–88.75)**		**(89.06–97.07)**		**(98.00–101.69)**		**(102.16–107.4)**		**(107.86–111.86)**		**(112.17–117.1)**		**(117.87–125.42)**		**(125.73–228.66)**	
	***n***	**Min; Max Mean (SD)**	***n***	**Min; Max Mean (SD)**	***n***	**Min; Max Mean (SD)**	***n***	**Min; Max Mean (SD)**	***n***	**Min; Max Mean (SD)**	***n***	**Min; Max Mean (SD)**	***n***	**Min; Max Mean (SD)**	***n***	**Min; Max Mean (SD)**	***n***	**Min; Max Mean (SD)**	***n***	**Min; Max Mean (SD)**
UHDRS Motor	680	0; 64	648	0; 92	655	0; 97	719	0; 93	522	0; 97	697	0; 95	577	0; 116	700	0; 108	687	0; 107	694	3; 120
		3.1 (6.7)		7.3 (11.9)		12.8 (14.8)		20.4 (16.2)		26.9 (16.6)		31.9 (18.1)		38.1 (19.4)		43.1 (20.3)		49.0 (19.9)		60.5 (23.4)
UHDRS Motor / DCL	682	0; 4	649	0; 4	656	0; 4	723	0; 4	526	0; 4	701	0; 4	585	0; 4	702	0; 4	688	0; 4	699	0; 4
		0.6 (1.1)		1.3 (1.5)		2.3 (1.7)		3.1 (1.4)		3.5 (1.1)		3.8 (0.8)		3.9 (0.6)		3.9 (0.4)		4.0 (0.3)		4.0 (0.2)
UHDRS FAS	666	0; 25	629	0; 25	639	0; 25	705	3; 25	512	0; 25	685	0; 25	568	0; 25	688	0; 25	668	0; 25	685	0; 25
		24.6 (1.8)		23.8 (3.0)		23.2 (3.5)		22.1 (4.0)		21.0 (4.7)		19.7 (5.6)		18.1 (6.2)		16.5 (6.6)		14.9 (7.0)		11.0 (7.7)
UHDRS TFC	679	0; 13	650	1; 13	655	0; 13	723	0; 13	525	0; 13	700	0; 13	586	0; 13	702	0; 13	686	0; 13	699	0; 13
		12.6 (1.3)		12.0 (2.0)		11.4 (2.4)		10.5 (2.8)		9.8 (3.0)		9.1 (3.2)		8.0 (3.5)		7.2 (3.5)		6.4 (3.6)		4.7 (3.5)
UHDRS Independence Scale	682	60; 100	649	20; 100	655	20; 100	722	25; 100	524	20; 100	701	20; 100	584	5; 100	700	10; 100	688	5; 100	700	5; 100
		98.5 (5.2)		95.9 (9.6)		92.8 (11.2)		88.6 (12.8)		85.0 (13.6)		81.4 (15.3)		76.6 (17.2)		73.1 (16.9)		69.0 (18.5)		57.5 (22.3)
TUG (total time)	347	0.5; 34	313	3; 55	299	3.6; 49	350	1; 66	227	4.7; 45	290	3.5; 112	215	4; 45	238	4; 47	218	4.8; 45	163	5; 98
		7.8 (2.8)		8.6 (4.3)		8.7 (3.7)		9.6 (5.2)		9.8 (4.1)		10.2 (7.5)		11.2 (5.6)		11.7 (5.6)		12.7 (6.0)		16.2 (12.2)
SWRT (total correct)	679	25; 148	639	0; 155	651	0; 164	716	0; 177	519	0; 125	678	0; 123	559	0; 199	666	0; 120	649	0; 105	619	0; 100
		95.7 (18.9)		89.2 (22.3)		81.3 (23.3)		71.6 (22.4)		64.7 (21.1)		60.6 (22.4)		54.1 (22.1)		49.7 (20.8)		44.7 (20.9)		34.3 (22.5)
SDMT (total correct)	677	0; 87	643	0; 92	648	0; 101	709	0; 76	514	0; 85	674	0; 61	542	0; 63	645	0; 57	613	0; 51	560	0; 90
		53.4 (12.3)		47.4 (14.1)		40.6 (14.4)		33.9 (13.4)		29.1 (12.7)		26.0 (12.6)		21.3 (11.5)		19.1 (10.8)		16.6 (10.6)		11.9 (10.7)
SIT (total correct)	639	0; 92	591	0; 93	602	0; 100	661	0; 65	468	0; 92	607	0; 74	482	0; 78	558	0; 93	523	0; 69	438	0; 50
		45.6 (11.6)		41.8 (12.8)		37.0 (13.0)		31.8 (11.9)		28.3 (10.9)		25.9 (11.2)		22.4 (10.7)		20.4 (10.9)		18.8 (10.9)		15.4 (9.7)
VFT category (total correct)	679	2; 43	641	0; 45	649	0; 37	716	2; 55	521	0; 40	686	0; 32	569	0; 26	679	0; 30	667	0; 26	645	0; 28
		22.1 (5.9)		20.4 (6.2)		18.5 (6.0)		16.3 (6.3)		14.5 (5.8)		13.1 (5.5)		11.5 (5.2)		10.6 (5.1)		9.7 (5.2)		7.6 (4.9)
SCNT (total correct)	678	26; 121	637	0; 136	650	0; 113	716	0; 130	517	0; 103	680	0; 95	561	0; 100	668	0; 100	656	0; 80	627	0; 88
		75.5 (15.2)		69.9 (18.3)		62.9 (18.7)		55.1 (17.9)		48.7 (17.1)		46.2 (17.2)		40.5 (16.7)		37.3 (15.7)		33.8 (15.4)		27.1 (17.3)
Trail Making Test Part A (time to complete)	588	10; 240	528	3; 240	529	13; 240	562	4; 240	393	14; 240	501	14; 240	379	21; 240	460	18; 240	401	17; 240	323	23; 240
		25.9 (17.2)		29.7 (22.2)		37.3 (27.5)		47.5 (33.4)		53.7 (34.6)		62.3 (44.0)		73.4 (48.1)		85.5 (55.2)		99.4 (65.0)		121.5 (71.8)
Trail Making Test Part B (time to complete)	587	13; 240	526	16; 240	524	20; 240	556	10; 240	391	28; 240	486	28; 240	368	32; 240	436	31; 240	371	17; 240	293	45; 240
		52.2 (30.8)		64.3 (42.3)		85.9 (58.0)		107.0 (62.9)		126.6 (66.4)		141.4 (69.7)		159.0 (68.3)		174.1 (66.8)		184.9 (63.4)		205.1 (54.6)
VFT letters (total correct)	584	3; 84	529	0; 83	540	0; 73	583	0; 77	402	0; 71	540	0; 67	407	0; 61	478	0; 64	460	0; 65	380	0; 53
		40.7 (13.0)		38.9 (14.8)		34.4 (14.0)		29.1 (13.9)		26.9 (13.8)		23.4 (13.1)		20.4 (12.2)		19.1 (11.9)		16.6 (11.7)		12.7 (9.8)
MMSE	495	15; 30	438	12; 30	438	0; 30	480	12; 30	343	10; 30	454	11; 30	351	2; 30	407	7; 30	373	0; 30	330	0; 30
		28.8 (1.7)		28.2 (2.3)		27.7 (3.1)		26.9 (2.9)		26.6 (3.1)		25.8 (3.6)		24.9 (4.1)		24.5 (4.5)		23.5 (5.1)		21.7 (6.1)
PBA-s apathy	681	0; 16	644	0; 16	655	0; 16	718	0; 16	524	0; 16	695	0; 16	582	0; 16	698	0; 16	678	0; 16	654	0; 16
		1.2 (2.6)		1.7 (3.3)		2.2 (3.8)		2.5 (3.8)		2.9 (4.0)		3.2 (4.3)		3.8 (4.7)		4.1 (4.8)		4.0 (5.0)		4.5 (5.3)
PBA-s depression	681	0; 41	647	0; 44	655	0; 41	719	0; 41	525	0; 44	696	0; 48	580	0; 34	695	0; 44	677	0; 41	649	0; 33
		4.4 (5.7)		5.5 (7.1)		5.6 (7.3)		5.7 (6.6)		5.5 (6.4)		5.2 (6.5)		5.0 (6.2)		5.2 (6.5)		4.5 (5.9)		3.7 (5.2)
PBA-s irritability/aggression	681	0; 28	646	0; 28	655	0; 32	720	0; 32	526	0; 25	700	0; 32	584	0; 28	700	0; 25	686	0; 32	688	0; 32
		2.2 (3.7)		2.9 (4.5)		3.0 (4.9)		3.3 (5.1)		3.3 (4.5)		3.4 (4.8)		3.0 (4.9)		3.2 (4.9)		3.2 (5.1)		3.3 (5.5)
PBA-s psychosis	682	0; 18	643	0; 32	655	0; 20	718	0; 20	525	0; 32	696	0; 24	582	0; 22	695	0; 16	672	0; 14	651	0; 24
		0.2 (1.1)		0.2 (1.7)		0.3 (1.6)		0.3 (1.7)		0.3 (1.9)		0.3 (1.6)		0.4 (1.9)		0.4 (1.7)		0.3 (1.4)		0.4 (2.1)
PBA-s executive functioning	678	0; 24	643	0; 32	653	0; 32	715	0; 28	524	0; 32	693	0; 32	579	0; 32	696	0; 32	676	0; 32	650	0; 32
		1.4 (3.3)		2.1 (4.4)		2.3 (4.4)		2.6 (5.0)		2.7 (5.0)		3.3 (5.3)		3.9 (5.9)		4.0 (6.0)		3.9 (5.9)		4.8 (6.5)
SF-12 physical functioning	546	25.7; 57.3	516	25.7; 57.3	491	25.7; 57.3	550	25.7; 57.3	388	25.7; 57.3	466	25.7; 57.3	390	25.7; 57.3	429	25.7; 57.3	419	25.7; 57.3	337	25.7; 57.3
		54.9 (5.9)		53.5 (7.3)		51.4 (9.1)		49.7 (10.0)		47.8 (10.1)		46.3 (11.0)		44.7 (11.5)		42.2 (11.7)		40.6 (11.5)		37.5 (11.9)
SF-12 mental health	545	18.9; 63	514	18.9; 63	493	18.9; 63	549	18.9; 63	386	18.9; 63	465	18.9; 63	386	18.9; 63	427	18.9; 63	416	18.9; 63	337	18.9; 63
		50.3 (9.3)		48.4 (10.1)		48.4 (10.2)		47.8 (10.5)		48.4 (10.1)		48.7 (10.2)		49.2 (10.0)		50.2 (9.9)		49.7 (10.6)		51.0 (11.1)
HADS-SIS depression	461	0; 17	447	0; 21	426	0; 19	465	0; 18	330	0; 18	401	0; 20	322	0; 17	354	0; 21	337	0; 21	257	0; 21
		3.4 (3.4)		4.3 (4.2)		5.2 (4.4)		5.3 (4.0)		5.9 (4.0)		5.7 (4.3)		5.6 (4.0)		6.1 (4.2)		6.1 (4.1)		6.2 (4.4)
HADS-SIS anxiety	462	0; 19	448	0; 21	426	0; 21	469	0; 21	331	0; 19	401	0; 20	325	0; 20	355	0; 18	336	0; 20	256	0; 21
		5.7 (4.1)		6.3 (4.3)		6.7 (4.6)		6.2 (4.2)		6.1 (4.1)		6.0 (4.2)		5.6 (4.1)		5.3 (4.0)		5.4 (4.0)		4.7 (3.9)
HADS-SIS irritability	461	0; 19	449	0; 23	426	0; 24	461	0; 24	332	0; 20	401	0; 23	322	0; 17	355	0; 19	338	0; 22	257	0; 21

#### Motor

For Total Motor Score (TMS), extreme skewness of data was observed for the three lowest CAP score deciles (encompassing CAP scores of 26 through 89), with density plots clearly illustrating floor effects in the lowest deciles. At higher deciles, data resembled a more normal distribution, although flattened curves with non-pronounced peaks were observed, indicating a somewhat even distribution of scores across the observed range, underscored by kurtosis statistics (not shown). A similar pattern was observed for SF-12 physical functioning, with data in the highest CAP deciles resembling a near uniform distribution across the scale. For Timed Up and Go (TUG), data demonstrated extreme skew across the full disease spectrum. *Post-hoc* sensitivity analyses were performed for TUG, given the observation of outliers, illustrated in Figure 3. Several outlier removal thresholds were explored, the most extreme of which was a maximum threshold of 40 s, resulting in the removal of 20 data points from 2,660 total observations (i.e., 0.75% of data). Imposition of this threshold had a major impact on skewness statistics and data distribution relative to the original distribution observed; data resembled a somewhat normal distribution from CAP decile 2 on (see Supplementary Figures 1, 2). Diagnostic Confidence Level (DCL) data were reasonably distributed up to and including CAP decile 4 (encompassing CAP scores of 26 through 97). Beyond this point, data became increasingly skewed as CAP score increased. Density plots provide a clear illustration of pronounced ceiling effects in the advanced phases of the disease (Figure 3), as does the percentage of individuals scoring the maximum score on this outcome (Supplementary Table 1).

#### Function

For the functional assessments, i.e., Functional Assessment Score (FAS), Total Functional Capacity (TFC), and Independence Scale (IS), extreme skewness of data was also observed at lower CAP score deciles, indicative of ceiling effects in the early stages of the HD life-course, also clearly observed in the corresponding density plots. From decile 4 on (encompassing CAP scores of 89 and above), data began to resemble a more normal distribution, verging on uniform at the higher end of the spectrum (Figures 2, 3).

#### Cognition

Except for the Trail Making tests and The Mini Mental State Examination (MMSE), all other cognitive assessments examined demonstrated a relatively normal distribution of scores within each CAP score decile (Figures 2, 3).

*Post-hoc* sensitivity analyses were performed for Symbol Digit Modality Test (total correct) and Stroop Interference Test (total correct) given the observation of six valid but implausible zero scores in the two initial CAP deciles (SDMT: *n* = 4; SIT: *n* = 2). Removal of these values had a negligible effect on the skewness statistics and did not alter conclusions (Supplementary Figure 3).

Trail Making Test Part A demonstrated extreme data skew up to and including CAP decile 6 (CAP scores ≤ 107), with clear floor effects apparent in the very lowest deciles, while Part B demonstrated extreme data skew in the 2 lowest deciles only (CAP scores ≤79). MMSE data were heavily skewed in the lowest deciles, with clear ceiling effects apparent in the corresponding density plots, while a more normal data distribution was observed from decile 4 onwards (CAP scores of ≥89).

#### Psychiatric/Behavioral

For the Hospital Anxiety and Depression Scale—Snaith Irritability Scale (HADS-SIS) depression, anxiety and irritability subscales, data distribution was relatively normal with broad variation across all CAP score deciles, indicating an absence of floor/ceiling effects at all disease stages. The same was true of Short Form Health Survey−12v2 (SF-12) Mental Health.

Most Problem Behaviors Assessment—Short (PBA-s) scores exhibited extreme data skew across the disease spectrum, of which the psychosis scale was the most extreme example. PBA-s depression was an exception (data were skewed but within acceptable range across almost all deciles), as was PBA-s apathy, which also demonstrated skew but within an acceptable range from decile 3 onwards (CAP scores ≥80).

## Discussion

Using data from Enroll-HD, a large and diverse observational study of HD, we examined distributions of scores on several well-established HD clinical assessments for evidence of instrument constraints, specifically floor/ceiling effects. These results are an important addition to existing clinimetric data for the outcome measures analyzed, which is limited, particularly for populations in the prodromal phase of the disease.

Most assessments demonstrated good utility (i.e., absence of floor/ceiling effects) across CAP deciles 5 through 10, equivalent to the period spanning clinical diagnosis/onset through to the most severe stages of the disease examined. The only exceptions to this rule were DCL, which (unsurprisingly) demonstrated poor utility from CAP decile 5 onwards (encompassing CAP score of 100, approximating onset), and assessments which demonstrated poor utility ubiquitously. Conversely, many assessments demonstrated poor utility in the lower CAP deciles, equivalent to the very earliest phases of the disease life course, prior to diagnosis/onset. This was true of the motor assessments, all functional assessments, and the two Trail Making Tests from the cognitive domain, indicating that instruments that are sensitive to the very earliest premanifest changes in motor and function performance are required. These are already under development (31, 32).

In contrast, all the cognitive assessments (bar the Trail Making Tests and MMSE) and all the HADS-SIS subscales demonstrated good utility across the full disease life course.

However, certain assessments demonstrated poor utility across the full disease spectrum, including most PBA-s scales and TUG (although removal of extreme outliers from the TUG data suggests this task does show promise in the earliest stages of the HD life-course). This should be further explored, perhaps with digital versions of the task using smartphone sensors, as is currently being used in the Parkinson's disease field (33).

A thorough understanding of the clinimetric properties of assessments, including instrument constraints, is imperative in conducting robust and meaningful research. If there is no (or very limited) variability in a specific outcome measure in a cohort due to assessment constraints, then using that outcome to assess a candidate drug may lead researchers to conclude that the candidate has no effect on disease. This may, or may not, be true. For example, use of FAS as an outcome in a clinical trial targeting early premanifest HDGECs would be inadvisable given the floor effects observed in scores in this phase of the disease.

Here we describe the utility of commonly used HD clinical assessments by disease stage, for consideration when designing clinical studies and/or trials. We also expand on what is known with regards to psychiatric/behavioral assessments. Our results in relation to the PBA-s outcomes, which were highly skewed across most deciles, imply low utility of this assessment across the HD spectrum. In contrast, the HADS-SIS depression, anxiety and irritability outcomes demonstrated a superior clinimetric performance, consistent with previous conclusions that HADS is a more appropriate assessment than PBA-s for the constructs mentioned (34). Note that we do *not* comment on the utility of these assessments to track disease progression.

The extremely large and diverse nature of the Enroll-HD data sample affords us confidence in the robustness of our results. This is further bolstered by the rigorous data quality control procedures implemented within Enroll-HD, from point of data entry through to onsite and remote data review, designed to maximize data integrity. We do, however, acknowledge several limitations. First, our approach to the identification of instrument constraints, specifically floor/ceiling effects, was relatively simple, based principally on assessment of data skewness in conjunction with other basic descriptive statistics. Other complimentary methods may be applied to further and more comprehensively assess the presence and severity of floor/ceiling effects. Second, our consideration of both “core” and “extended” assessments from the Enroll-HD protocol assessment battery resulted in a somewhat limited sample size for certain assessments, given the optional nature of the extended measures. Third, we acknowledge that our results may have been mildly affected by missing data, should the reason for missingness relate to disease stage/assessment score. This is plausible for both optional assessments (identified in Table 2), as well as assessments requiring active participation (e.g., TUG) where more advanced individuals who have progressed further may be unable (or may not be asked) to attempt these tasks. If such situations have occurred, the most extreme/worst scores would have been masked. Fourth, we concede that our approach to disease staging was non-standard; we used a quantile grouping method to approximate equivalency of group size for statistical reasons, but acknowledge that this resulted in categories encompassing widely differing ranges of CAP score, with the widest categories noted at the lowest and highest deciles. A more standard approach may have aided interpretation and application of results and afforded increased resolution in identifying the boundaries of floor/ceiling effects in the earliest and latest phases of disease. Nevertheless, it is well-established that a CAP score of 100 coincides approximately with the occurrence of clinical diagnosis and it is therefore relatively simple to infer which deciles encompass prodromal and manifest phases of the disease. Fifth, we highlight that in assessments with no set maximum scale threshold (e.g., TUG, or assessments where the vast majority score within a limited scale range, e.g., PBA psychosis) the presence of even a few extreme scores (plausible outliers) can dramatically influence skewness statistics, leading to inaccurate conclusions regarding floor/ceiling effects. Consequently our conclusions regarding these specific assessments should be considered with caution.

We applied a simple analytical approach to data ascertained from a very large and diverse clinical cohort to examine constraints of commonly used HD assessments as a function of disease stage. Most assessments demonstrated good utility from onset onwards, others across the full disease life course, while others performed poorly across the spectrum. Investigations to develop instruments sensitive to subtle differences in performance in earlier stages of the disease spectrum are warranted.

## Data Availability Statement

Publicly available datasets were analyzed in this study. This data can be found at: https://www.enroll-hd.org/for-researchers/access-data/.

## Ethics Statement

Ethical review and approval was not required for the study on human participants in accordance with the local legislation and institutional requirements. Written informed consent from the participants' legal guardian/next of kin was not required to participate in this study in accordance with the national legislation and the institutional requirements.

## Author Contributions

DA: conception, organization, execution of research project, design, execution, review and critique of statistical analysis, writing of the first draft, review, and critique of manuscript preparation. JW: conception, organization, execution of research project, design, review and critique of statistical analysis, writing of the first draft, review, and critique of manuscript preparation. NG-K and BL: conception, organization, execution of research project, review and critique of statistical analysis, writing of the first draft, review and critique of manuscript preparation. CF-A: conception and organization research project, review and critique of statistical analysis, review, and critique of manuscript preparation. RL: conception, organization, execution of research project, execution of statistical analysis, review, and critique of manuscript preparation. AF: conception of research project, design, and execution of statistical analysis. OH, KA, and JS: conception, organization, execution of research project, review, and critique of manuscript preparation. CS: conception, organization, execution of research project, design, review and critique of statistical analysis, writing of the first draft, review, and critique of manuscript preparation. All authors contributed to the article and approved the submitted version.

## Conflict of Interest

NG-K is member of the Enroll-HD Clinical Trials Committee, CHDI. KA received $2,000 payment for her involvement in the Enroll-HD Scientific Oversight Committee. She has also received travel funding to attending Enroll-HD SOC meetings, including those where this manuscript was planned and discussed. Through Georgetown University, KA also receives funding from CHDI as site principal investigator for the Georgetown MedStar Enroll-HD clinical site. CF-A was an employee at CHDI Management and was employed by company ClinMed LLC. BL has served on Scientific Advisory Boards for sRNAlytics, the Huntington's Disease Society of America, and the Huntington Society of Canada. JS is the chair of the Scientific Oversight Committee of Enroll-HD, for which she receives an annual stipend of $2,000 from CHDI Foundation. Through Monash University, JS also receives funding from CHDI as site principal investigator of Monash University/Calvary Health Care Bethlehem Hospital Enroll-HD clinical site. CS and JW are employed by CHDI Management, Inc. as advisors to CHDI Foundation, Inc. CHDI Foundation, Inc. is a non-profit biomedical research organization exclusively dedicated to collaboratively developing therapeutics that substantially improve the lives of individuals with Huntington's disease. The remaining authors declare that the research was conducted in the absence of any commercial or financial relationships that could be construed as a potential conflict of interest.

## Footnotes

^1^John H. Warner JDL, Mills JA, Langbehn DR, Ware J, Mohan A, Sampiao C. *Standardizing the CAP Score in Huntington's Disease I: Predicting Age-at-Onset* (in preparation) (2020).

## References

[1] Walker FO. Huntington's disease. Lancet. (2007) 369:218–28. 10.1016/S0140-6736(07)60111-1 17240289

[2] Paulsen J Langbehn DR Stout JC Aylward E Ross CA Nance M. Detection of Huntington's disease decades before diagnosis: the Predict-HD study. J Neurol Neurosurg Psychiatry. (2008) 79:874–80. 10.1136/jnnp.2007.128728 18096682 PMC2569211

[3] Rodrigues FB Abreu D Damásio J Goncalves N Correia-Guedes L Coelho M. Survival, mortality, causes and places of death in a European Huntington's disease prospective cohort. Mov Disord Clin Pract. (2017) 4:737–42. 10.1002/mdc3.12502 30363513 PMC6174515

[4] Guha M. APA Dictionary of Statistics and Research Methods. Reference Reviews (2014).

[5] Landwehrmeyer GB Fitzer-Attas CJ Giuliano JD Gonçalves N Anderson KE Cardoso F. Data analytics from Enroll-HD, a global clinical research platform for Huntington's disease. Mov Disord Clin Pract. (2017) 4:212–24. 10.1002/mdc3.12388 30363395 PMC6174428

[6] Enroll-HD (2015). Available online at: https://www.enroll-hd.org (accessed 11 January, 2020).

[7] UHDRS Copyright© Huntington Study Group. UHDRS Total Motor Score 0-124 (1999).

[8] UHDRS Copyright© Huntington Study Group. UHDRS Diagnostic Confidence Level 0-4 (1999).

[9] UHDRS Copyright© Huntington Study Group. UHDRS Total Functional Capacity 0-13 (1999).

[10] UHDRS Copyright© Huntington Study Group. UHDRS Functional Assessment Scale 0-25 (1999).

[11] UHDRS Copyright© Huntington Study Group. UHDRS Independence Scale 5-100 (1999).

[12] Podsiadlo D Richardson S. The timed “Up & Go”: a test of basic functional mobility for frail elderly persons. J. Am Geriatr Soc. (1991) 39:142–8. 10.1111/j.1532-5415.1991.tb01616.x 1991946

[13] Western Psychological Services. SDMT copyright ©: Western Psychological Services Los Angeles (1973, 1976, 1982).

[14] Lezak MD Howieson DB Loring DW Fischer JS. Neuropsychological Assessment. Oxford: Oxford University Press (2004).

[15] Stroop Color Naming Test. ENROLL-REQ-2003-EN-1.0.3 Enroll-HD 1.0 Data Validation Requirements (2019).

[16] Stroop Word Reading Test. ENROLL-REQ-2003-EN-1.0.3 Enroll-HD 1.0 Data Validation Requirements (2019).

[17] Stroop Interference Test. ENROLL-REQ-2003-EN-1.0.3 Enroll-HD 1.0 Data Validation Requirements (2019).

[18] Maximum time for administration as indicated in. ENROLL-REQ-2003-EN-1.0.3 Enroll-HD 1.0 Data Validation Requirements (2019).

[19] Folstein MF Folstein SE McHugh PR. “Mini-mental state”: a practical method for grading the cognitive state of patients for the clinician. J Psychiatr Res. (1975) 12:189–98. 10.1016/0022-3956(75)90026-6 1202204

[20] Depression; Suicidality; Anxiety. ENROLL-REQ-2003-EN-1.0.3 Enroll-HD 1.0 Data Validation Requirements (2019).

[21] Irritability; Anger. ENROLL-REQ-2003-EN-1.0.3 Enroll-HD 1.0 Data Validation Requirements (2019).

[22] Delusions; Hallucinations; Disoriented Behavior. ENROLL-REQ-2003-EN-1.0.3 Enroll-HD 1.0 Data Validation Requirements (2019).

[23] Apathy. ENROLL-REQ-2003-EN-1.0.3 Enroll-HD 1.0 Data Validation Requirements (2019).

[24] Perseveration; OCD. ENROLL-REQ-2003-EN-1.0.3 Enroll-HD 1.0 Data Validation Requirements (2019).

[25] Snaith R Zigmond A. Hospital anxiety and depression scale (HADS). Acta Psychiatr Scand. (1994) 67:361–70. 10.1111/j.1600-0447.1983.tb09716.x 6880820

[26] Snaith R Constantopoulos A Jardine M McGuffin P. A clinical scale for the self-assessment of irritability. Br J Psychiatry. (1978) 132:164–71. 10.1192/bjp.132.2.164 623950

[27] Ware JE Kosinski M Keller SD Lincoln R. I. How to Score the SF-12 Physical and Mental Summary Scales. Boston, MA: The Health Institute, New England Medical Center (1995).

[28] Shoulson I Fahn S. Huntington disease: clinical care and evaluation. Neurology. (1979) 29:1. 10.1212/WNL.29.1.1 154626

[29] Prentice GR. Understanding and using statistics in psychology. Irish J Psychol. (2009) 30:226. 10.1080/03033910.2009.10446313

[30] West SG Finch JF Curran PJ. Structural equation models with nonnormal variables: Problems and remedies (1995).

[31] ClinicalTrials.gov. FuRST 2.0 Cognitive Pre-Testing. (2016). Updated August 8, 2017. Available online at: https://ClinicalTrials.gov/show/NCT02881931 (accessed May 17, 2021).

[32] Fuller R Feigenbaum P Fitzer-Attas C La Pelle N Luo S Sampaio C. FuRST2. 0: Development of a modified functional rating scale for use in premanifest and early-manifest Huntington's disease. Mov Disord. (2018) 33. Available online at: https://www.mdsabstracts.org/abstract/furst2-0-development-of-a-modified-functional-rating-scale-for-use-in-premanifest-and-early-manifest-huntingtons-disease/ (accessed June 28, 2021).

[33] Lipsmeier F Taylor KI Kilchenmann T Wolf D Scotland A Schjodt-Eriksen J. Evaluation of smartphone-based testing to generate exploratory outcome measures in a phase 1 Parkinson's disease clinical trial. Mov Disord. (2018) 33:1287–97. 10.1002/mds.27376 29701258 PMC6175318

[34] Mestre TA van Duijn E Davis AM Bachoud-Lévi AC Busse M Anderson KE. Rating scales for behavioral symptoms in Huntington's disease: critique and recommendations. Mov Disord. (2016) 31:1466–78. 10.1002/mds.26675 27296904

