# Supplementary material for: Utility of Huntington's Disease Assessments by Disease Stage: Floor/Ceiling Effects
Source: Front Neurol. 2021 Jul 15;12:595679. doi: 10.3389/fneur.2021.595679 (PMC8320772; doi:10.3389/fneur.2021.595679)

**
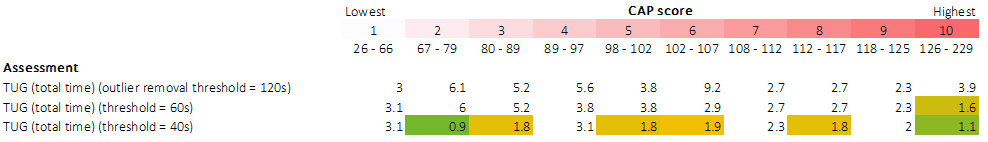
**

**Supplementary Figure 1. Skewness of Timed Up and Go data by CAP score: sensitivity analyses.** The impact of outlier removal threshold on skewness statistics for TUG by CAP score decile was examined. The original threshold of 120 seconds resulted in removal of 2 observations (from a total of 2,660); the 60 second threshold resulted in the removal of 8 observations; the 40 second threshold resulted in the removal of 20 observations. A threshold of +/- 2, indicating extreme positive or negative skew, was defined *a priori* to identify extreme skew, indicative of floor/ceiling effects. Cells with values more extreme than these thresholds are colored white. All remaining cells, with skewness statistics ranging from -2 to +2, are color coded on a yellow-green-yellow gradient, centered at 0 (green), indicative of a perfect normal distribution of data, graduating to yellow as values become more extreme.

**
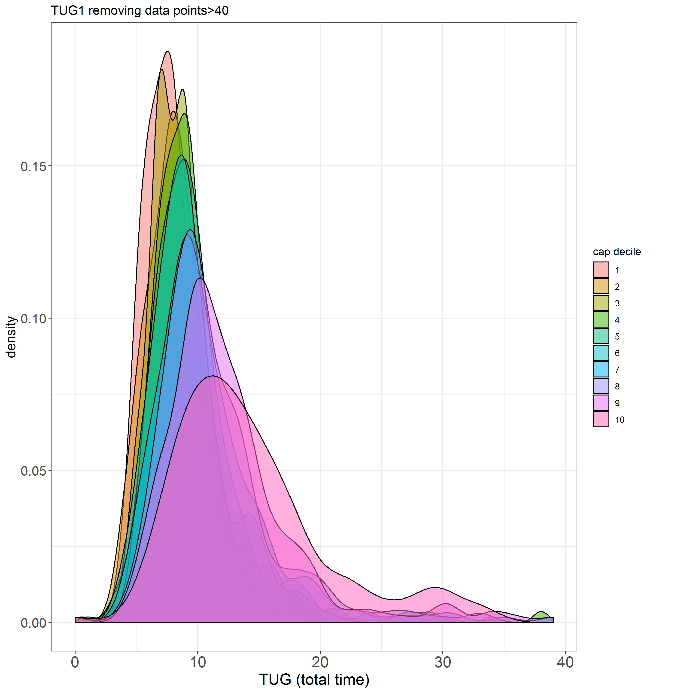
**

**Supplementary Figure 2. Density plot of Timed Up and Go data by CAP score: sensitivity analysis.** Density plot based on TUG data with an outlier removal threshold of 40 seconds. This threshold resulted in the removal of 20 observations from 2,660 total (i.e., 0.75% of data).

**Supplementary Figure 3. Skewness of Symbol Digit Modality Test (total correct) and Stroop Interference Test (total correct) by initial CAP score: sensitivity analyses.** The impact of zero removal on skewness statistics for SDMT (total correct) for the initial CAP score deciles was examined. After removing all zero values observed in CAP deciles 1 and 2 for SDMT (n=4 observations removed) and SIT (n=2 observations removed), the skewness statistics were mildly impacted, but our original conclusions remain unchanged, i.e., a relatively normal distribution of scores were observed for these assessments across each CAP decile group.

**Supplementary Table 1:** Percentage of participants scoring minimum and maximum scores within each CAP score decile for each assessment.


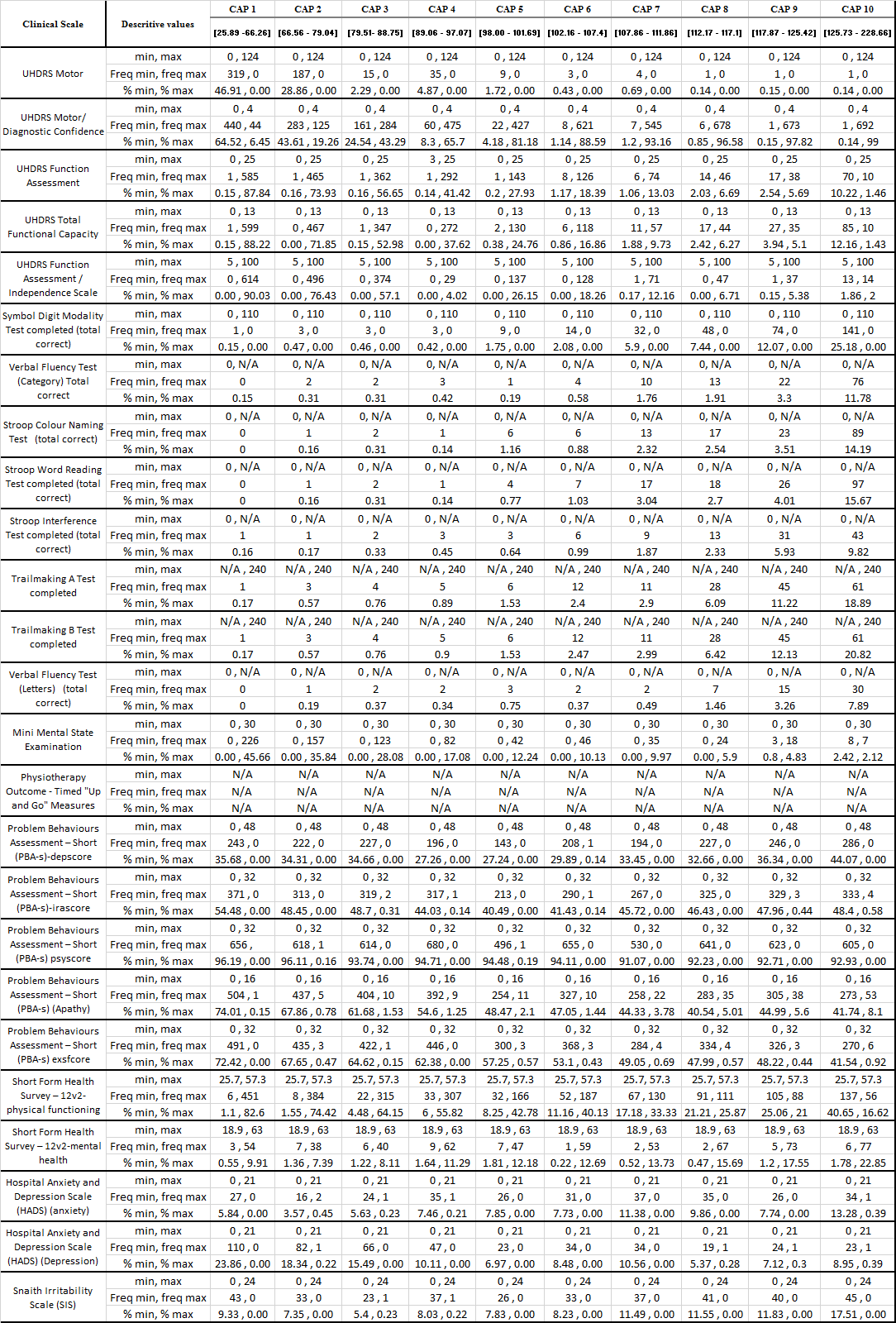

Supplement: Supplementary file 1 [file Data_Sheet_1.docx]
